# New Appliances and Things Medical

**Published:** 1907-06-01

**Authors:** 


### NEW APPLIANCES AND THINGS MEDICAL.

**[We shall be glad to receive at our Office, 28 & 29 Southampton Street, Strand, London, W.C., from the manufacturers, specimens of all new preparations and appliances which may be brought out from time to time.]**

### PEPSALIA.

(Cerebos, Limited, Newcastle-on-Tyne, and 3 Maiden Lane, London, E.C.)

Pepsalia consists of a white powder which contains common salt, imparting to it a distinct saline taste; and also pepsine, the proteolytic ferment of the gastric juice in a dry state. The pepsine is associated with an acid. Thus in the preparation before us we have, to all intents and purposes, a means of not only satisfying our taste for saltness, but also reinforcing our digestive power. That there are many cases of so-called atonic dyspepsia which are relieved by the administration of pepsine is an undoubted clinical fact, and we regard pepsalia as a most convenient form in which to prescribe pepsine, as it can be used as an ordinary table salt.

### LYXHAYR.

(Lyxhayr, Limited, Grove Mills, Mitcham, Surrey.)

This is a vegetable fibre, which the manufacturers claim is the cleanest and healthiest material for bedding and stuffing purposes, as it is odourless, and has been sterilised, and so rendered perfectly hygienic. It appears to be a good substitute for hair, and presents special advantages which make it popular for hospital use. With the object of testing it thoroughly and arriving at a practical conclusion as to the merits of this article, we have made arrangements to keep a Lyxhayr mattress under observation while used in a hospital ward, and so soon as it has been long enough in use to justify a definite opinion we shall publish a full report of the experiment and its results.

